# SARS-CoV-2 (MA10) Infection Aggravates Cerebrovascular Pathology in Endothelial Nitric Oxide Synthase-Deficient Mice

**DOI:** 10.3390/v17060784

**Published:** 2025-05-29

**Authors:** Saifudeen Ismael, Meenakshi Umar, Blake Ouvrier, Gregory Hall, McKenzie Cummins, Arjun Sapkota, Grant Talkington, Amanda Louise White, Richard Milner, Damir B. Khismatullin, Gregory Bix

**Affiliations:** 1 Department of Neurosurgery, Clinical Neuroscience Research Center, School of Medicine, Tulane University, New Orleans, LA 70112, USA; sismael@tulane.edu (S.I.); mumar@tulane.edu (M.U.); bouvrier@tulane.edu (B.O.); ghall9@tulane.edu (G.H.); gtalkington@tulane.edu (G.T.); awhite29@tulane.edu (A.L.W.); 2 Tulane Brain Institute, Tulane University, New Orleans, LA 70112, USA; 3 Department of Biomedical Engineering, Tulane University, New Orleans, LA 70118, USA; ecummins@tulane.edu (M.C.); damir@tulane.edu (D.B.K.); 4 San Diego Biomedical Research Institute, San Diego, CA 92121, USA; asapkota@sdbri.org (A.S.); rmilner@sdbri.org (R.M.); 5 Department of Neurology, School of Medicine, Tulane University, New Orleans, LA 70112, USA; 6 Department of Microbiology and Immunology, School of Medicine, Tulane University, New Orleans, LA 70112, USA; 7 School of Public Health and Tropical Medicine, Tulane University, New Orleans, LA 70122, USA

**Keywords:** SARS-CoV-2, endothelial nitric oxide synthase, vascular dementia, neuroinflammation, senescence

## Abstract

SARS-CoV-2 can cause neurological issues, including cognitive dysfunction in COVID-19 survivors. Endothelial dysfunction, a key mechanism in COVID-19, is also a risk factor for vascular dementia (VaD). Reduced nitric oxide (NO) bioavailability is a pathogenic factor of endothelial dysfunction and platelet aggregation in COVID-19 patients, and endothelial NO synthase (eNOS) levels decline with advancing age, a risk factor for both COVID-19 morbidity and VaD. SARS-CoV-2 also induces cellular senescence and senescence-associated secretory phenotype (SASP). We hypothesized that eNOS deficiency would worsen neuroinflammation, senescence, blood–brain barrier (BBB) permeability, and hypercoagulability in eNOS-deficient mice. Six-month-old eNOS^+/−^ (pre-cognitively impaired experimental VaD) and wild-type (WT) male mice were infected with mouse-adapted (MA10) SARS-CoV-2. Mice were evaluated for weight loss, viral load, and markers of inflammation and senescence 3 days post-infection. eNOS^+/−^ mice showed more weight loss (~15%) compared to WT mice (~5%) and increased inflammatory markers (*Ccl2*, *Cxcl9*, *Cxcl10*, *IL-1β*, and *IL-6*) and senescence markers (p53 and p21). They also exhibited higher microglial activation (Iba1) and increased plasma coagulation and BBB permeability, despite comparable lung viral loads and absence of virus in the brain. This is the first experimental evidence demonstrating that eNOS deficiency exacerbates SARS-CoV-2-induced morbidity, neuroinflammation, and brain senescence, linking eNOS to COVID-19-related neuropathology.

## 1. Introduction

Although SARS-CoV-2 infection causes an acute and long-term broad spectrum of clinical manifestations, its pathophysiology is still under investigation [1,2]. SARS-CoV-2 causes a variety of neurological sequelae in COVID-19 survivors, including fatigue and cognitive dysfunction [3]. While the pulmonary system is considered the primary target for acute SARS-CoV-2 infection, accumulating evidence suggests that it also affects the vasculature in extrapulmonary organs by inducing thrombotic vascular disease, endothelial dysfunction, and multi-organ injury [4]. COVID-19 caused vascular inflammation, senescence, endothelial dysfunction, coagulopathies, and impaired fibrinolysis [5,6,7]. To date, mounting evidence supports that endothelial dysfunction is the central mechanism of COVID-19 illness and a major risk factor for vascular dementia (VaD) [3,8]. Endothelial dysfunction stems, in part, from an imbalance between nitric oxide (NO) generated by the endothelial nitric oxide synthase (eNOS) and reactive oxidant species (ROS) produced by uncoupled eNOS [9,10]. Clinical observations suggest that reduced NO levels are associated with acute SARS-CoV-2 morbidity [11,12,13], and sustenance of vascular NO levels improved clinical outcomes in patients with severe disease [14,15,16]. However, the association between eNOS deficiency and SARS-CoV-2 infection has not been evaluated.

The NO produced by eNOS maintains vascular homeostasis by maintaining blood flow. NO also mediates anti-inflammatory responses by preventing leukocyte adhesion, platelet activation, and aggregation [12,17], thereby reducing thromboinflammation. Reduction in NO levels induces platelet aggregation and reduces fibrinolysis by inhibiting tissue-type plasminogen activator (t-PA) [17,18]. Consistently, dysregulated plasma coagulation and fibrinolytic activity were detected in COVID-19 patients [19,20]. SARS-CoV-2 spike protein binds with fibrin, forming prothrombotic clots, and mediates thromboinflammatory response [21]. Conversely, fibrin-rich intravascular microthrombi were detected in the cerebral cortex of eNOS-deficient mice [22]. However, the association between SARS-CoV-2 infection and eNOS reduction has not been thoroughly evaluated. We hypothesize that eNOS deficiency aggravates neuroinflammation, blood–brain barrier (BBB) permeability, and brain senescence after SARS-CoV-2 infection. To develop an effective SARS-CoV-2 infection model, we utilized a novel mouse-adapted SARS-CoV-2 (MA10) virus, which recapitulates clinical COVID pathology and neuroinflammation in wild-type (WT) mice. SARS-CoV-2 (MA10) was generated by serial passage of the SARS-CoV-2 Wuhan strain in mice [23]. Seven substitution mutations enable them to access murine ACE2 (mACE2) with less preference for human ACE2. Since the MA10 strain can utilize mouse ACE2, it eliminates the need for transgenic mice expressing human ACE2 in experimental studies [23]. Our lab has shown that MA10 in infected wild-type animals exhibits acute and chronic brain changes (e.g., inflammation and BBB permeability) [24,25]. Here, we investigated whether eNOS deficiency contributes to pulmonary and cerebrovascular endothelial dysfunction following SARS-CoV-2 infection in eNOS-deficient mice. WT C57Bl/6 and eNOS^+/−^ mice (since there is no complete deficiency of eNOS reported in humans, eNOS^+/−^ mice were used over eNOS^−/−)^ were infected with SARS-CoV-2 (MA10) to determine whether eNOS deficiency worsens acute COVID-19 morbidity, neuroinflammation, and senescence and promotes endothelial dysfunction.

## 2. Materials and Methods

### 2.1. SARS-CoV-2 Infection

In this study, 6-month-old C57BL/6J (WT) and eNOS^+/−^ mice (The Jackson Laboratory, Bar Harbor, ME, USA) were housed in the animal facility at Tulane University School of Medicine in standard humidity (45–50%), temperature (21–25 °C), and 12 h light cycle with food and water ad libitum. All experimental procedures, including sample handling, inactivation, and removal from BSL3 containment, were conducted in compliance with the regulations of the Institutional Animal Care and Use Committee of Tulane University. Mice were infected intranasally with either mock (PBS) or 1 × 10^4^ plaque-forming units (pfu) of SARS-CoV-2 (MA10) strain of severe acute respiratory syndrome coronavirus 2 (SARS-CoV-2, BEI Resources, Manassas, VA, USA, NR-55329) in 50 µL by ABSL3-trained staff as previously described [23,24,26]. Animals were evaluated daily for clinical signs of illness and changes in body weight. The mice were euthanized at 3 days post-infection (3 dpi) by CO_2_ asphyxiation followed by cervical dislocation. The mouse lungs and brains were harvested for gene expression and histological analysis by immunofluorescence according to standard protocol.

### 2.2. Gene Expression

Brain and lung tissue were homogenized in TRIzol™ Reagent (Invitrogen, Carlsbad, CA, USA, 15596-026), and RNA was extracted according to RNA extraction kit manufacturer instructions (RNeasy Plus Mini Kit; Qiagen, Germantown, MD, USA, 74136). Furthermore, 1 μg of RNA was reverse transcribed to cDNA using iScript reverse transcriptase master mix (Bio-Rad, Hercules, CA, USA). Gene expression analysis was carried out with QuantStudio 3 Real-Time PCR Systems (Life Technologies, Carlsbad, CA, USA) using TaqMan PCR Master Mix and premixed primers/probe sets (Thermo Fisher Scientific, Waltham, MA, USA) specific for *Il-1β* (Mm00434228_m1), *Il6* (Mm00446190_m1), *Ccl2* (Mm00441242_m1), *Cxcl10* (Mm00445235_m1), *Cxcl9* (Mm00434946_m1), *Vcam1* (Mm01320970_m1), *p21* (Mm04207341_m1), *p53* (Mm01731290_g1), *p16* (Mm00494449_m1), and *Gapdh* (Mm99999915_g1, control gene) (Thermoscientific) for gene expression as previously described (36680154). The mRNA expression of *Gapdh* was used as a housekeeping gene to normalize target gene expression. For the determination of genomic (N) and subgenomic mRNA encoding the N gene (sgm-N), quantification was performed as previously described [27,28].

### 2.3. Immunofluorescence

Brain tissues were fixed in 10% phosphate-buffered formalin, paraffin-embedded, and sliced into 5 μm thickness. Brain sections were deparaffinized in a series of xylene and rehydrated through a descending grade of ethanol, followed by heat-induced antigen retrieval with Tris-based antigen retrieval solution (pH 8). The slides were washed with PBS with 0.3% Triton X-100 and blocked with 10% normal goat serum for 1 h. Primary antibodies anti-Iba1 (1:1000, Cat# 019-19741, FUJIFILM Wako Pure Chem Corp, Osaka, Japan), SARS-nucleocapsid, and fibrinogen (1:100, Cat# 341552, Sigma, St. Louis, MO, USA) were prepared in blocking buffer and incubated overnight at 4 °C. The endothelial cells were labeled with lectin 488 (DL-1174-1, Vector Laboratories, Newark, CA, USA). Slides were then incubated with either goat anti-rabbit IgG secondary antibody, Alexa Fluor™ 594 (1:500; cat#: A-11037), or Alexa Fluor™ 488 (1:500; cat#: A-11008, Thermo Fisher Scientific, Shanghai, China) for 60 min at room temperature (RT) in normal goat serum following washing with PBS. After washing in PBS, mount with ProLong Gold Antifade media with DAPI to label the nuclei. Sections were imaged using a Nikon Eclipse Ti-S/L100 microscope with a 20×/0.8 NA objective (Nikon, Tokyo, Japan). Laser settings and detector gains were kept constant for all samples. Images were analyzed with ImageJ 1.53k (NIH), and fluorescence intensity was quantified after background subtraction. Negative controls omitting primary antibodies were included in all experiments.

### 2.4. Integrated Quasi-Static Acoustic Tweezing Thromboelastometry (i-QATT) for Coagulation Analysis

The i-QATT was used to measure clot firmness and turbidity [29] in our in vivo studies. This technique provides comprehensive coagulation analysis with a single drop of blood (4–6 microliters in volume, ~100 times less than the blood sample volume in TEG/ROTEM and plasma tests) and without blood sample exposure to artificial surfaces [30]. Whole blood samples were collected at 3 dpi, and plasma was prepared at the ABSL3 laboratory. Anticoagulated virus-inactivated plasma samples were recalcified and exposed to the extrinsic pathway activator (thromboplastin-D) prior to i-QATT coagulation analysis.

### 2.5. Statistics

Statistical tests were performed using GraphPad Prism, version 9.3.1 (GraphPad Software, San Diego, CA, USA). Data are presented as mean ± SEM. Significant differences were designated using one-way ANOVA followed by Tukey’s multiple comparison test. Statistical significance was taken at the *p* < 0.05 level.

## 3. Results

### 3.1. Effect of SARS-CoV-2 (MA10) Infection on Body Weight, Genomic and Subgenomic Viral Load in eNOS^+/−^ Mice

**Figure 1:**
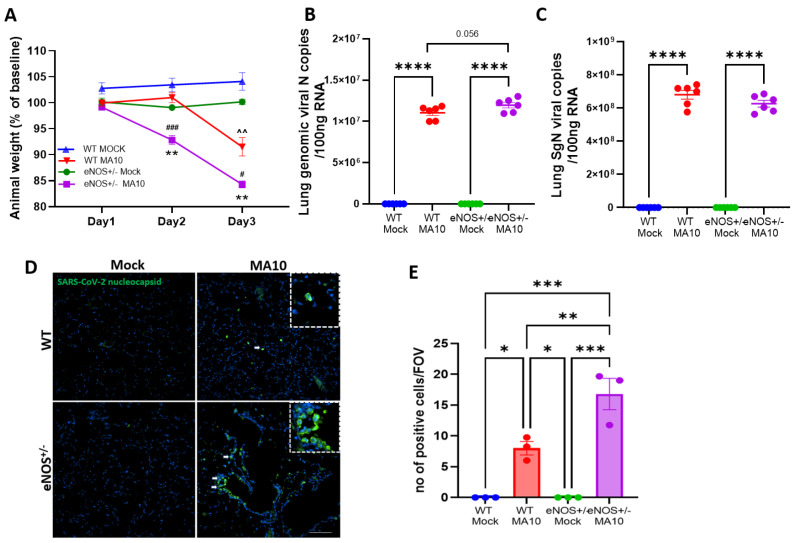
eNOS^+/−^ mice show intense disease severity compared to WT mice following mouse-adapted SARS-CoV-2 (MA10) infection. Six-month-old male eNOS^+/−^ and WT mice were inoculated with 1 × 10^4^ pfu via the intranasal route with mock (PBS) or MA10 strain of SARS-CoV-2. (**A**) MA10 infection induced a more significant decrease in body weight in eNOS^+/−^ mice compared to infected WT mice. ^^ *p* < 0.01 vs. WT Mock, ^###^ *p* < 0.001 vs. WT MA10, ^#^ *p* < 0.05 vs. WT MA10, ** *p* < 0.01 vs. eNOS^+/−^ Mock. Upon 3 days post-infection (3 dpi), mice were euthanized, and RNA was isolated from the lung and brain samples and analyzed for (**B**) genomic (N) and (**C**) subgenomic (SgN) viral copies in the lungs. There was no significant difference in genomic or subgenomic viral copies between MA10-infected WT and eNOS^+/−^ mice. Immunofluorescence analysis of (**D**,**E**) viral nucleocapsid showed that there were more positive cells in the lungs of eNOS^+/−^ mice than WT mice following infection. Data are represented as mean ± SEM, * *p* < 0.05, ** *p* < 0.01, *** *p* < 0.001, **** *p* < 0.0001, *n* = 3–6/group. Scale bar = 100 µm.

In this study, 6-month-old eNOS^+/−^ and WT mice were infected with 10^4^ pfu of SARS-CoV-2 (MA10), and mice were evaluated daily for changes in body weight. While no mortality was observed in any of the infected groups, a significant decrease in body weight was observed in WT and eNOS^+/−^ mice at 3 dpi following MA10 infection (Figure 1A) compared to mock-infected WT and eNOS^+/−^ mice (*p* < 0.001). However, we observed significantly more weight loss in eNOS^+/−^ mice at 3 dpi than in WT MA10-infected mice (*p* < 0.05). Notably, eNOS^+/−^ mice, but not WT mice, started losing weight as early as 2 dpi (*p* < 0.001). To determine the viral copies in the brain and lungs, total (N) and subgenomic (SgN) viral load were detected. Although there was a significant increase in pulmonary genomic and subgenomic viral load (Figure 1B,C) in the infected groups as compared to mock-infected mice (*p* < 0.001), as expected, only genomic viral copy was slightly increased in the infected eNOS^+/−^ group compared to infected WT mice (*p* < 0.056). The SgN were identical in both infected groups (Figure 1C). Immunofluorescence analysis showed that there were more nucleocapsid-positive cells in the lungs of infected eNOS^+/−^ mice (Figure 1D,E) compared to WT mice (*p* < 0.01). However, there were no detectable viral copies of both SgN and N and nucleocapsid in the brain (Supplementary Figure S1) regardless of genotype.

### 3.2. MA10 Induces Pulmonary Cytokine and Chemokine Responses in eNOS^+/−^ Mice and WT Mice

**Figure 2:**
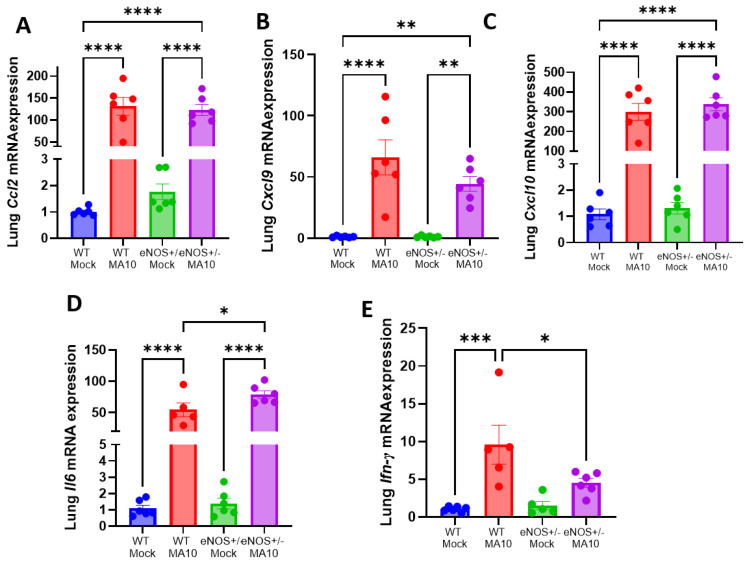
MA10 infection increases pulmonary proinflammatory response in eNOS^+/−^ mice more than in WT. MA10 infection induced a significant increase in (**A**) *Ccl2*, (**B**) *Cxcl9*, (**C**) *Cxcl10*, (**D**) *Il6*, and (**E**) Ifn-γ mRNA in WT and eNOS^+/−^ mice compared to WT mock mice at 3 dpi. Data are represented as mean ± SEM, * *p* < 0.05, ** *p* < 0.01, *** *p* < 0.001, **** *p* < 0.0001, *n* = 5–6/group.

To determine MA10-induced pulmonary inflammation, the mRNA expression of C-C motif ligand 2 (*Ccl2*), Interleukin-6 (*IL6*), C-X-C motif chemokine ligand 9 (*Cxcl9*), and C-X-C motif chemokine ligand 10 (*Cxcl10*) were evaluated by quantitative real-time PCR. We found that the mRNA levels of *Ccl2*, *Il6*, *Cxcl9*, and *Cxcl10* were significantly elevated (*p* < 0.001) by MA10 infection in WT and eNOS^+/−^ mice (Figure 2A–C). The mRNA expressions of *Ccl2*, *Cxcl9*, and *Cxcl10* were not different between MA10-infected WT and eNOS^+/−^ mice. However, the expression of *Il6* (Figure 2D) was significantly increased in MA10-infected eNOS^+/−^ mice compared to infected WT mice (*p* < 0.05). Interestingly, the expression of antiviral protein interferon (IFN)-γ (Figure 2E) was significantly elevated in WT mice (*p* < 0.01) but only trended upward in eNOS^+/−^ mice following MA10 infection to a level that was significantly less (*p* < 0.05) than it was in infected WT mice.

### 3.3. eNOS^+/−^ Mice Showed Increased Neuroinflammatory Response Following MA10 Infection

**Figure 3:**
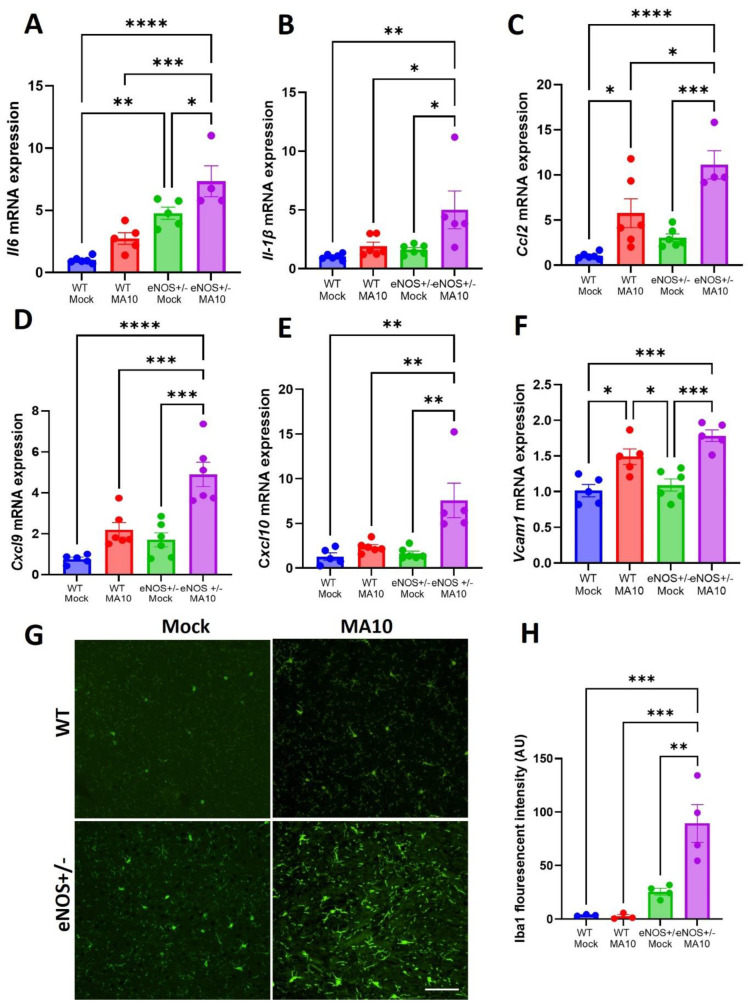
eNOS^+/−^ mice showed an increased neuroinflammatory response compared to WT mice following MA10 infection. (**A**) *Il-6*, (**B**) *Il1β*, (**C**) *Ccl2*, (**D**) *Cxcl9*, (**E**) *Cxcl10*, and (**F**) *Vcam1* mRNA expression in the brain at 3 dpi with MA10 or mock. Immunofluorescence analysis of Iba1-positive signal in the prefontal cortex (**G**,**H**) of eNOS^+/−^ and WT mice. MA10 infection induced increased *Il-6*, *Il1β*, *Ccl2*, *Cxcl9*, *Cxcl10*, and *Vcam1* mRNA expression and microglial activation in eNOS^+/−^ mice > WT mice, as also compared to WT mock and eNOS^+/−^ mock mice, respectively. Scale bar = 50 µM (20×). Data are presented as mean ± SEM. *p* values represent mock vs. SARS-CoV-2 (MA10) challenged groups *n* = 4–5/group; * *p* < 0.05. ** *p* < 0.01, *** *p* < 0.001, **** *p* < 0.0001.

Real-time PCR analysis of mRNA from whole brain tissue revealed elevated expression of proinflammatory mediators *ll-6*, *Il-1β*, *Ccl2*, *Cxcl9*, and *Cxcl10* (Figure 3A–E) in both the WT and eNOS^+/−^ mice 3 days post-MA10 infection. Multiple comparison analysis showed that mRNA expression of *Il-6*, *Il-1β*, *Ccl2*, *Cxcl9*, and *Cxcl10* (Figure 3A–E) was significantly elevated in eNOS^+/−^ mice compared to infected WT mice at 3 dpi. Furthermore, the expression of vascular cell adhesion molecule-1 (*Vcam1*) mRNA (Figure 3F), an endothelial activation marker, was significantly elevated in WT (*p* < 0.05) and eNOS^+/−^ mice following MA10 infection. It was further increased in eNOS^+/−^ mice than in WT mice following MA10 infection, although this increase was not statistically significant. In addition, immunofluorescence analysis showed increased Iba1 fluorescent intensity (Figure 3G,H) in eNOS^+/−^ mice compared to infected WT mice (*p* < 0.01).

### 3.4. eNOS^+/−^ Mice Showed Increased Brain p53/p21 Senescence Pathway Following MA10 Infection

**Figure 4:**
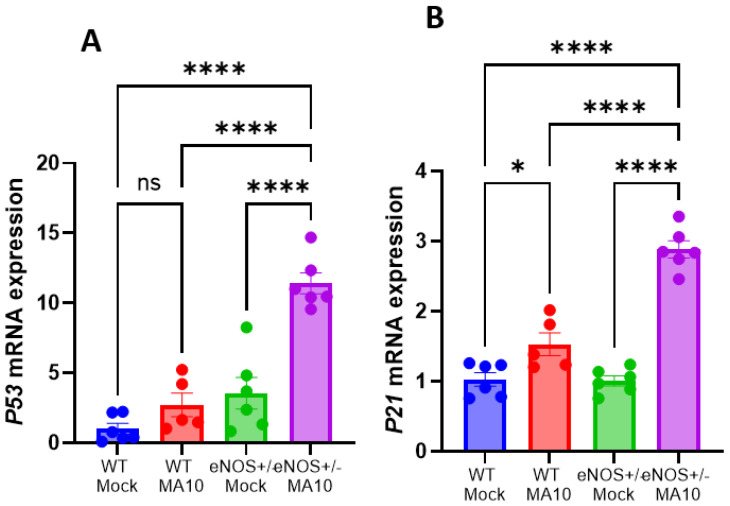
MA10 infection increases brain senescence in eNOS^+/−^ mice compared to WT. (**A**) MA10 infection-induced p53 mRNA is significantly higher in eNOS^+/−^ mice compared to MA10-infected WT and WT and eNOS^+/−^ mock mice. (**B**) The expression of p21 mRNA is significantly increased in MA10-infected WT and eNOS^+/−^ mice compared to WT and eNOS^+/−^ mock mice. MA10 significantly increased p53 mRNA in infected eNOS^+/−^ mice compared to WT MA10-infected mice. Mean ± SEM, * *p* < 0.05, **** *p* < 0.0001, ns, not significant, *n* = 5–6/group.

Since virus-induced senescence (VIS) was detected in COVID patients [31], we evaluated the expression of various cellular senescence markers such as *p21* and *p53* by real-time PCR. The mRNA expressions of *p21* and *p53* (Figure 4A,B) were increased by MA10 infection in both WT and eNOS^+/−^ mice. Interestingly, eNOS^+/−^ mice showed significantly more increased expression of *p21* and *p53* mRNA than WT mice following MA10 infection. Increased p21 and p53 mRNA expression along with various proinflammatory levels indicates clear activation of senescence and senescence-associated secretory phenotype (SASP) in eNOS^+/−^ mice after MA10 infection.

### 3.5. eNOS^+/−^ Mice Showed Increased Coagulatory Pathway Activation Following MA10 Infection

**Figure 5:**
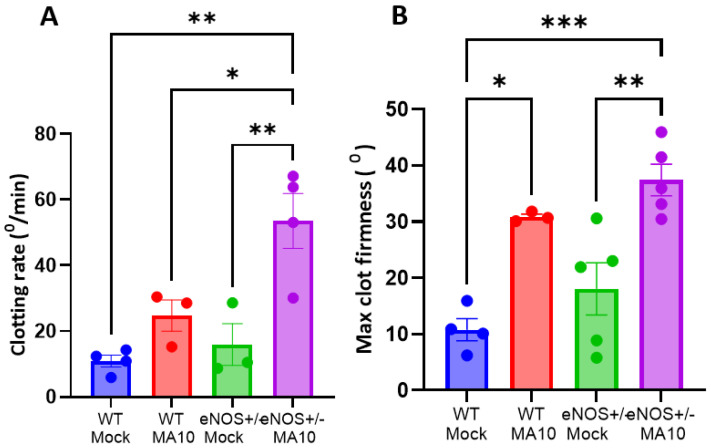
MA10 induces hypercoagulability in eNOS^+/−^ mice. Plasma analysis using the i-QATT technique showed that (**A**) clotting rate and (**B**) maximum clot firmness are elevated in MA10-infected eNOS^+/−^ mice compared to infected WT mice. Data are mean ± SEM. * *p* < 0.05, ** *p* < 0.01, *** *p* < 0.001, *n* = 3–5/group.

i-QATT analysis of plasma samples at 3 dpi showed that the rate of coagulation (Figure 5A) is elevated in MA10-infected eNOS^+/−^ mice compared to uninfected eNOS^+/−^ and WT mice and was further elevated than WT MA10-infected mice. In addition, clot firmness (Figure 5B), an indicator of functional fibrinogen level, was elevated in eNOS^+/−^ > infected WT mice.

### 3.6. eNOS^+/−^ Mice Showed Increased BBB Permeability Following MA10 Infection

**Figure 6:**
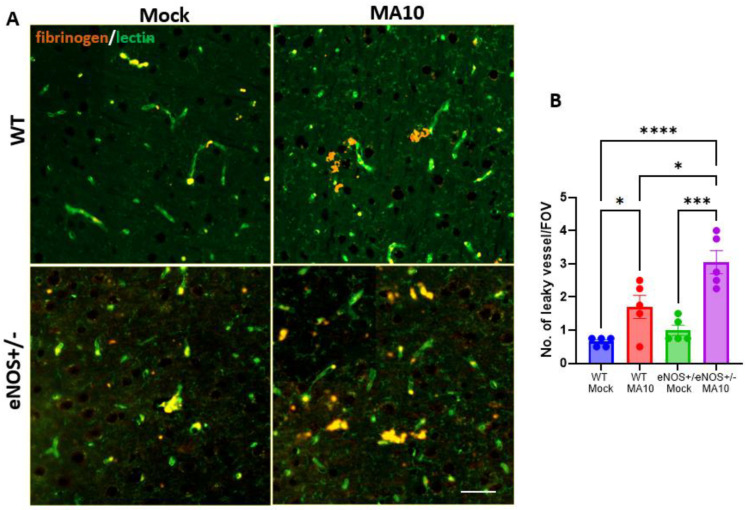
MA10 infection increases BBB permeability in eNOS^+/−^ mice more than in WT mice. Six-month-old male eNOS^+/−^ and WT mice were inoculated with SARS-CoV-2. (**A**) MA10. (**B**) IF analysis of fibrinogen (orange) and lectin (green). Quantification of fibrinogen leak showed that MA10 induced more vascular leak in eNOS^+/−^ than in WT mice. Data are mean ± SEM. * *p* < 0.05, *** *p* < 0.001, **** *p* < 0.0001 *n* = 5/group. Scale bar = 100 µm.

To evaluate BBB permeability, brain sections were immunostained for fibrinogen and lectin (endothelial cells). MA10 infection increased brain fibrin/fibrinogen leak in the prefrontal cortex, eNOS^+/−^ > WT (Figure 6).

## 4. Discussion

eNOS (also known as NOS 3) is normally expressed in endothelial cells, where it produces vasoactive NO from L-arginine that controls vascular homeostasis by maintenance of blood pressure and anti-atherosclerotic effects [32,33]. In addition, the antiviral properties of NO are well studied [34,35]; NO effectively prevented SARS-CoV-2 viral replication in vitro mediated by the inhibition of S-nitrosation of 3SARS-CoV-2 3CL cysteine protease, which is required for viral replication [36]. Clinical evidence suggests that the reduced concentration of venous erythrocytic iron-nitrosyl complex (HbNO), a marker of NO bioavailability, in patients with severe COVID-19 results in elevated oxidative stress and pro-thrombotic and inflammatory responses [13]. Consequently, NO inhalation emerges as a possible therapeutic approach that could increase patients’ oxygenation and improve clinical outcomes [11,14,15]. A randomized clinical trial demonstrated that oral administration of L-arginine, a substrate for NO synthase, in adults with severe COVID-19 significantly reduced the need for respiratory support and, foremost, the duration of hospitalization [37]. In addition, treatment with the NO donor, S-nitroso-N-acetylpenicillamine, reduced viral replication and cytopathological changes in epithelial cells in vitro [16]. Furthermore, plasma from COVID-19 hospitalized patients reduced NO production in cultured human aortic endothelial cells [38], and interaction between Spike protein and endothelial ACE2 impaired eNOS activity [39]. These observations suggest that decreased NO bioavailability appears as a likely pathogenic factor of cerebrovascular dysfunction in COVID-19 patients. In addition, eNOS deficiency causes various neuropathologies in mice; partial eNOS deficiency also causes progressive hypoperfusion, cerebral amyloid angiopathy, BBB disruption, white matter damage, and cognitive impairment, eventually leading to VaD [22]. The administration of sodium nitrate in drinking water ameliorated white matter damage, and sensory motor function in eNOS-deficient mice [40].

In this study, we investigated the effect of eNOS deficiency on SARS-CoV-2 MA10 pathogenesis. We found that MA10 increased infection-associated disease severity in eNOS^+/−^ mice as indicated by increased weight loss, exacerbated neuroinflammatory responses, increased BBB permeability, and dysregulated coagulation pathway activation despite no difference in pulmonary pathology. In vitro evidence showed increased neurotrophism of SARS-CoV-2 towards CNS cells such as neurons and astrocytes [41,42]. However, in line with previous observations [43,44], MA10 viral RNA was not detected in the infected brain, implying that the neuropathological events are independent of brain viral invasion. Systemic inflammatory response and alteration in adaptive and innate immune response are well-established features of SARS-CoV-2 infection [42]. Elevated systemic inflammation induces endothelial activation and increased BBB permeability [45,46]. This will allow the infiltration of immune cells and inflammatory mediators into the central nervous system, where it activates resident microglia and contributes to neuroinflammation and brain pathology even in the absence of direct viral infection [47]. As previously demonstrated [24], MA10 infection elevated transcriptomic levels of proinflammatory markers in the lungs and brain. Pulmonary inflammation is comparable in eNOS^+/−^ mice, whereas neuroinflammation is significantly higher in MA10-infected eNOS^+/−^ mice than in WT mice.

Recently, VIS was identified in SARS-CoV-2 pathology [48]. VIS is induced by many species of viruses, including members of the Polyomaviridae, Retroviridae, Paramyxoviridae, Rhabdoviridae, Parvoviridae, and Coronaviridae families [49,50,51,52,53,54]. Senescence is a cellular response to multiple stresses, such as injury and aging, where cells enter irreversible cell cycle arrest and acquire a senescence-associated secretory phenotype (SASP). The SASP involves secretion of various proinflammatory cytokines [55] and has a pathological role in SARS-CoV-2-associated neurovascular morbidity [56]. Experimental evidence supports that SARS-CoV-2 infection induces pulmonary senescence and SASP activation [7,57]. Further, RNA sequencing analysis of postmortem frontal cortex samples revealed an upregulation of aging-related pathways in the COVID-19 cases compared to age-matched controls [31]. Endothelial cells are the first cells to undergo senescence with aging [58], and reduced eNOS activity is the hallmark of endothelial senescence and dysfunction [59,60]. Conversely, inhibition of eNOS activity causes increased senescence in endothelial cells [61,62]. However, the contribution of eNOS in SARS-CoV-2-induced cellular senescence is not yet evaluated. Our data showed that MA10 induced brain senescence in WT and eNOS^+/−^ mice, as indicated by increased expression of *p21* and *p53* mRNA. Interestingly, mRNA expression of both markers was further elevated in eNOS^+/−^ mice than in infected WT mice, indicating that eNOS deficiency exacerbated MA10-induced brain senescence. *p53* is a transcriptional regulator protein that increases the expression of cell cycle inhibitor *p21*. *P21* induces senescence by inhibiting the cyclin-mediated phosphorylation of retinoblastoma (RB) [63]. However, further studies are necessary to evaluate their protein level expression and to identify the cellular localization of these markers.

A number of clinical and experimental studies reported that BBB damage is associated with SARS-CoV-2 infection, and increased BBB permeability induces neuroinflammation and cognitive dysfunction. Partial eNOS deficiency also caused cerebral BBB leakage and smooth muscle degeneration in mice [22]. Intriguingly, we found that the combination of eNOS deficiency and MA10 infection exacerbated fibrinogen leak into the brain parenchyma in eNOS^+/−^ mice. Recent evidence suggests that SARS-CoV-2 induces BBB damage through dysregulation of endothelial Wnt/β-catenin signaling, and targeted stimulation of the Wnt signaling pathway restored the BBB function [64]. Intriguingly, β-catenin is a positive regulator of endothelial survival through stimulation of eNOS activity in human endothelial cells [65]. Future studies will investigate the potential role of endothelial Wnt/β-catenin signaling in MA10’s BBB-opening effects in eNOS^+/−^ mice.

SARS-CoV-2 infection is often associated with a hypercoagulability state, as demonstrated by activated platelets and neutrophil extracellular traps, resulting in increased risks of disease severity and mortality [66,67,68]. Our findings revealed that MA10 infection exacerbated clotting rate and clot firmness, a measure of fibrinogen level in eNOS^+/−^ mice as measured by the integrated quasi-static acoustic tweezing thromboelastometry. Elevated platelet activation was often observed in severe COVID-19 patients and is a critical predictor of mortality [66]. Consistently, dysregulated plasma coagulation and fibrinolytic activity were detected in COVID-19 patients [19,20]. SARS-CoV-2 spike protein binds with fibrin, forming prothrombotic clots, and mediates thromboinflammatory response [21]. Further, incubation of plasma samples collected from severe patients activated naïve platelets ex vivo, suggesting the pathological role of platelets in COVID-19. Conversely, NO is a vasoactive molecule with antithrombotic and anti-inflammatory activity that prevents leukocyte adhesion, platelet activation, and aggregation [12,17], thereby reducing thromboinflammation. Reduction in NO levels causes platelet aggregation and reduced fibrinolysis by inhibiting tissue-type plasminogen activator (t-PA) [17,18], which could be a possible explanation for the increased incidence of ischemic stroke in COVID-19 subjects. Conversely, fibrin-rich intravascular microthrombi were detected in the cerebral cortex of eNOS-deficient mice [22], confirming the pathological role of NO deficiency in hypercoagulopathy state independent of infection, which is a risk factor for ischemic stroke, myocardial infarction, and pulmonary embolism.

We acknowledge that we used the SARS-CoV-2 MA10 strain, a mouse-adapted variant of the original Wuhan isolate, and current SARS-CoV-2 variants mediate much less pathogenic response than the ancestral strain. However, recent studies have shown that the Delta and Omicron variants of SARS-CoV-2 can also induce neuropathological changes in mouse models [69,70]. These investigations used K18-hACE2 transgenic mice, which express human ACE2 under the K18 promoter in epithelial cells. While widely used, this model does not accurately reflect the natural expression pattern of the ACE2 receptor, limiting its utility in studying SARS-CoV-2-induced neuropathology. In contrast, the MA10 strain can infect wild-type laboratory mice such as BALB/c or C57BL/6 [24], enabling the study of disease progression in wild-type mice. MA10 infection results in acute lung injury and mortality, but with no detectable viral presence in the brain at the time of peak infection, indicating a more restricted neurotropism in this model and making it well-suited for investigating pulmonary and long-term post-viral outcomes in a more physiologically relevant context. We will use the MA10-R2G of the SARS-CoV-2 (MA10) variant [71], which may induce less mortality, for further studies.

The evidence provided indeed highlights a significant role for eNOS in modulating the severity of SARS-CoV-2-induced neuropathology. The use of the MA10 mouse-adapted strain offers a valuable model that closely mirrors key features of human COVID-19, particularly in terms of neuroinflammation and senescence. Although this is the first experimental evidence linking eNOS deficiency to neuropathology, brain senescence, and hypercoagulopathy induced by SARS-CoV-2 (MA10) infection, our study is limited by its acute duration (3 dpi). Further long-term studies are required to explore how MA10 infection might chronically impact cerebrovascular pathology and potentially accelerate the onset or worsen the severity of cognitive impairment in young or aged eNOS^+/−^ mice, respectively. However, our results suggest that maintaining or improving vascular nitric oxide levels could be a potential therapeutic strategy to alleviate COVID-19-associated neuropathology.

## Data Availability

All data generated or analyzed during this study are included in this published article and its Supplementary Information Files.

## Supplementary Materials

The following supporting information can be downloaded at: https://www.mdpi.com/article/10.3390/v17060784/s1. Figure S1: No virus was detected in the brain following mouse-adapted SARS-CoV-2 (MA10) infection.
