# Supplementary material for: SARS-CoV-2 (MA10) Infection Aggravates Cerebrovascular Pathology in Endothelial Nitric Oxide Synthase-Deficient Mice
Source: Viruses. 2025 May 29;17(6):784. doi: 10.3390/v17060784 (PMC12197800; doi:10.3390/v17060784)
Supplement: Supplementary file 1 [file viruses-17-00784-s001.zip › viruses-3599019-supplementary.pdf]

No virus was detected in the brain following mouse-adapted SARS-CoV2 (MA10) infection

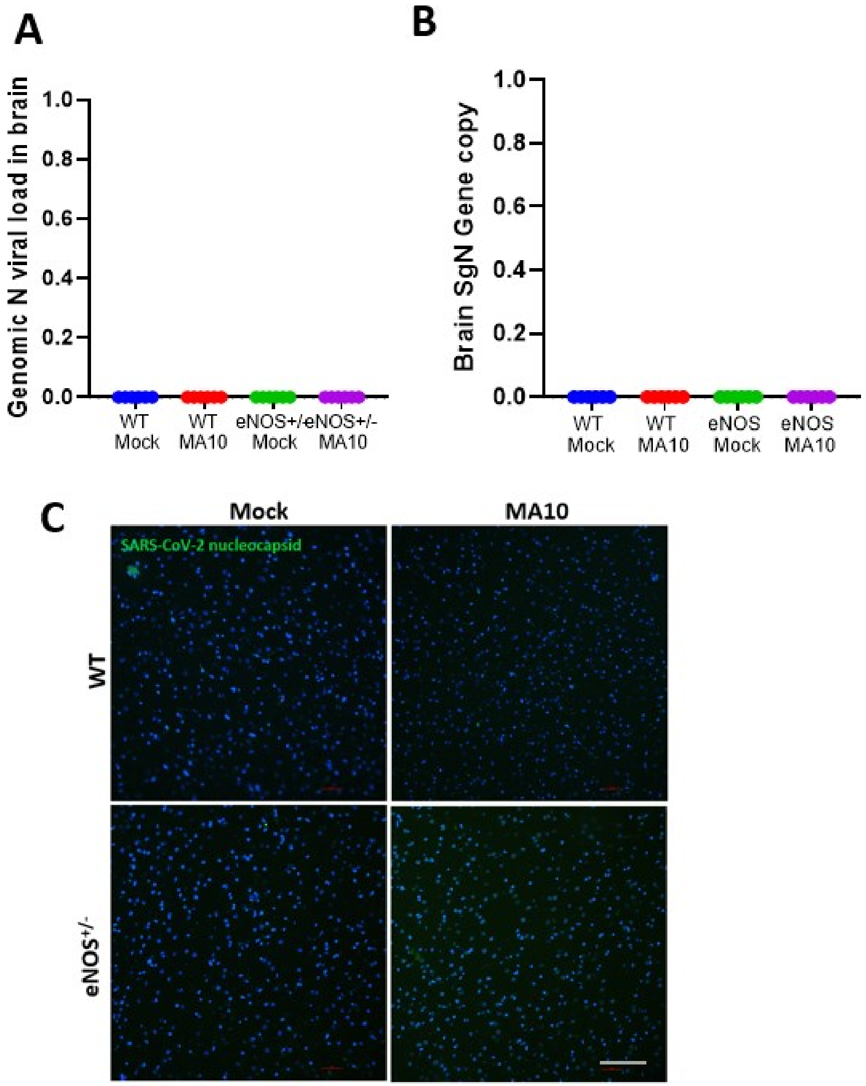

Figure S1. 6-month-old male eNOS+/ and WT mice were inoculated with $1x10^4$ pfu via the intranasal route with mock (PBS) or MA10 strain of SARS-CoV-2. Upon 3 days post-infection (3 dpi), mice were euthanized, and RNA was isolated from the lung and brain samples and analyzed for (A) Genomic (B) Subgenomic-N viral copies and (C) nucleocapsid in the brain. There was no viral copies or nucleocapsid were detected in MA10 infected WT and eNOS+/- mouse brain. Data represented as mean±SEM.
